# The Effect of Materialism on Impulsive Buying: The Mediating Role of the Diderot Effect

**DOI:** 10.3390/bs15101403

**Published:** 2025-10-16

**Authors:** Rana Şen Doğan

**Affiliations:** Department of Econometrics, Faculty of Economics and Administrative Sciences, Manisa Celal Bayar University, Manisa 45140, Türkiye; rana.dogan@cbu.edu.tr; Tel.: +90-2362018083

**Keywords:** materialism, impulsive buying, Diderot effect, digital consumption, consumer behavior, consumer’s emotions

## Abstract

Materialism is recognized as an important determinant of consumer behavior. However, studies exploring how materialism influences impulsive buying through the Diderot effect in digital contexts remain scarce. This study analyzes data from 416 adult consumers in Türkiye using Structural Equation Modeling (SEM). Four hypotheses were tested: (i) materialism increases the Diderot effect, (ii) the Diderot effect strengthens impulsive buying, (iii) materialism directly influences impulsive buying, and (iv) materialism indirectly affects impulsive buying through the Diderot effect. The findings support all four hypotheses. Materialism significantly increases both the Diderot effect and impulsive buying in online environments. The Diderot effect partially mediates this relationship and acts as an independent predictor of impulsive tendencies. The study also points out that digital stimuli such as recommendation systems, fast payment options, and social proof may reinforce this mechanism, highlighting the importance of understanding consumer behavior in digital settings. Overall, the results underline that materialistic desires, when combined with the completion motive of the Diderot effect, strongly shape impulsive tendencies. Beyond advancing theory, the study emphasizes that marketing strategies should go beyond promoting higher consumption and instead foster conscious and sustainable shopping practices. This approach not only supports consumer well-being, but also contributes to building ethical and sustainable markets.

## 1. Introduction

Digital commerce has transformed the way that consumers compare, evaluate, and purchase products. Engaging content, social proof, and personalized recommendations in online environments shorten decision-making time. When combined with easy payment options, the likelihood of impulsive buying increases. Personalized suggestions trigger identity and status-seeking motives, leading to purchases aligned with lifestyle (Shamu et al., 2024; Yang et al., 2025). The abundance of stimuli explains the high rates of unplanned and impulsive shopping in online environments (Açan et al., 2021; Gallin & Portes, 2024; Gillespie, 2023; Nyrhinen et al., 2024; TÜİK, 2024).

This study examines, in the context of digital commerce, the direct relationship between materialism and impulsive buying as well as the indirect relationship mediated by the Diderot effect, tested through Structural Equation Modeling (SEM). The study employs a cross-sectional design. Materialism is a value orientation linking possessions with identity and well-being (Belk, 1988; Mei et al., 2024; Richins, 2004). The Diderot effect refers to the “set completion” tendency following an initial acquisition, while impulsive buying (Li et al., 2025; McCracken, 1988; Qureshi et al., 2025) is an emotionally triggered, spur-of-the-moment purchase under limited reasoning (Feng et al., 2024; Rook, 1987). This framework is suitable for explaining how materialistic motives relate to “set completion” purchases under conditions of stimulus abundance and “one-click” payment options.

The accumulation of impulsive purchases may pave the way for compulsive cycles (Faraz & Anjum, 2025). The literature has associated materialism with “unplanned,” “impulsive,” or “compulsive” buying (Barbieri et al., 2025; Belk, 1988; Koul & Jasrotia, 2025; Richins, 2004). Here, “unplanned” refers only to the absence of pre-planning (Beatty & Ferrell, 1998); “impulsive” indicates sudden or emotionally triggered decisions under limited reasoning (Rook & Fisher, 1995); while “compulsive” denotes repetitive and uncontrollable compulsive behaviors (Faber & O’Guinn, 1989). This distinction has been maintained throughout the text. In this study, impulsive buying behavior is examined. Recent research has shown that the Diderot effect is not limited to unplanned purchases but is also associated with sudden and unforeseen impulsive behaviors (Santos et al., 2025).

Prior findings show that materialism increases the Diderot effect and that sensitivity to status/fit reinforces this tendency (Çakır & Eser, 2023; Ergenekon Arslan, 2022). The Diderot effect is positively associated with impulsive/unplanned purchasing; digital interfaces make complementary items salient, thereby strengthening this channel (Çakaröz et al., 2022; Santos et al., 2025). Evidence on set-based consumption conceptually supports this mechanism (Englis & Solomon, 1996). Moreover, the materialism → impulsive buying link is consistently positive across different samples (Atulkar & Kesari, 2018; Pupelis & Šeinauskienė, 2023; Raj et al., 2024). Taken together, this picture indicates a lack of studies that test the mediating role of the Diderot effect within a single integrated model in the digital context, underscoring the need for the present study.

Closing this gap moves beyond simple associations in the literature and seeks answers to the following questions: “Through which mediating mechanism does materialism influence impulsive purchasing in digital settings?”, “Is the Diderot effect truly a mediator?”, and “What is the practical digital counterpart of this mediation?” Empirically testing this mechanism allows researchers to partition the total effect into direct and indirect components and to quantify the share transmitted through the Diderot pathway, thereby making theory tests more rigorous and extensible. For practitioners, making the mechanism visible enables interventions to be targeted to the right context and calibrated precisely Linking design decisions to measurable principles reduces the risk of regret and disengagement caused by short-term aggressive configurations on digital platforms. As a result, returns and cart abandonment decline, satisfaction and repeat purchasing rise. In addition, resource use becomes more efficient, and trust is strengthened.

Within this framework, the Diderot effect is positioned as the mediating mechanism that explains the link between materialism and impulsive buying, and the following paths are tested: (i) materialism → Diderot effect, (ii) Diderot effect → impulsive buying, (iii) materialism → impulsive buying, (iv) materialism → Diderot effect → impulsive buying (indirect effect).

The study offers four contributions—theoretical, empirical, contextual, and managerial. Theoretically, the Diderot effect functions as a mediator: a first acquisition induces set-incongruity, amplifies completion motives, and accelerates choice; the mechanism is most salient in high-stimulus, low-friction settings. Empirically, the four paths are estimated within a single SEM, with direct and indirect effects obtained simultaneously; mediation is statistically significant and accounts for about 31% of the total effect, indicating that a substantial share operates through the mechanism. Contextually, the pattern is observed with one-click checkout, instant cart discounts, and “complete the look/customers also bought” prompts. Managerially, measures compatible with existing systems are outlined—capping recommendation frequency, adding a brief cart cool-off, providing a visible opt-out, and disclosing total cost before payment—which preserve user autonomy, reduce perceived steering, lower return/complaint rates, and strengthen perceptions of fairness and trust.

This study was conducted in Turkey. The Turkish sample provides an opportunity to observe the mechanism in a rapidly growing digital market. Integrated model findings on impulsive buying in Turkey remain limited; moreover, the effects of materialism on the Diderot effect and the role of the Diderot effect in impulsive buying have not been sufficiently explored. For example, Ergenekon Arslan (2022) proposed an integrated model of compulsive buying. Çakaröz et al. (2022) associated the Diderot effect with unplanned purchases, while Gürdin (2020) found no significant Diderot effect on general purchase decisions. This study seeks to clarify the mechanism within a cultural context. While the single-country focus may limit external validity, this limitation is addressed in the Section 5.4 (Limitations).

The theoretical and practical significance can be outlined as follows: the Diderot effect’s function of completing identity and style alignment suggests that mediation will be particularly visible in three contexts. First, in product categories that strongly reflect identity and enhance visibility; second, among consumers with low tolerance for inconsistency; and third, in digital environments where stimuli are intense and payment friction is low, such as with one-click checkout or instant cart discounts. Although these factors are not directly measured, they represent digital extensions of the mechanism. Thus, understanding the completion motive highlights how chain purchases are designed and where their limits lie.

Accordingly, this study makes the dynamics of digital marketing’s influence on consumer behavior theoretically visible, explaining the materialism–Diderot effect–impulsive buying relationship within a clarifying framework. In conclusion, the study contributes to balancing marketing practices while enhancing consumer awareness, thereby supporting healthier shopping habits, particularly in digital environments.

## 2. Theoretical Framework

People generally choose products that “fit” their identities (Sirgy, 1982). Products are often consumed as sets, complementing one another (Englis & Solomon, 1996). In the digital realm, however, the way a product is presented guides consumer decisions (Thaler & Sunstein, 2008). This study integrates these three points and positions the Diderot effect as the mediating link through which materialistic tendencies lead to impulsive buying. The following sections briefly define materialism, the Diderot effect, and impulsive buying, and summarize their theoretical connections based on the literature.

### 2.1. Materialism

From a materialistic perspective, consumption and material possessions are important means of enhancing subjective well-being and achieving life goals (Richins & Dawson, 1992). Therefore, for materialistic individuals, consumption is not merely a necessity but also an indicator of success and happiness (Belk, 1984; Richins, 2004; Ward & Walkman, 1971). For these individuals, possessions serve as markers of identity and status. The more they own, the more valuable they feel, placing possessions at the center of their lives (Belk, 1984; Richins & Dawson, 1992). Needs for social belonging and differentiation also fuel materialistic tendencies (Belk, 1984; Richins, 2004). Furthermore, self-control levels are linked to these tendencies. Specifically, materialistic individuals tend to have lower self-control and are more prone to spend easily (Vohs & Faber, 2007). Recent studies reveal that this tendency has become even more pronounced through digital platforms and social media (Jin & Ryu, 2024).

Concepts such as success, happiness, and centrality illustrate the multidimensional structure of materialism (Richins & Dawson, 1992). However, high correlations among these dimensions indicate that the construct does not always clearly differentiate (Manchiraju, 2013). For this reason, examining materialism as a single dimension encompassing these concepts offers a more holistic and robust approach (Hurst et al., 2013; Wong et al., 2011). Accordingly, in this study, materialism is treated as a unidimensional construct with a holistic perspective.

Materialism may drive individuals to spend continuously to enhance life satisfaction and feel more valuable. Over time, this process fosters unhealthy consumption habits. Indeed, materialism has been associated with impulsive and compulsive buying (Atulkar & Kesari, 2018; Dittmar & Bond, 2010; Ellyawati et al., 2024). These purchases manifest in different ways: seeking products that align with identity (Sirgy, 1982), constructing identity through brands (Escalas & Bettman, 2005), or preferring complementary products as a set (Englis & Solomon, 1996). Findings indicate that materialism intensifies the search for identity and coherence (Dittmar & Bond, 2010), which in turn lays the groundwork for the Diderot effect.

### 2.2. Diderot Effect

The Diderot effect offers an important perspective for understanding consumer behavior (McCracken, 1988; Santos et al., 2025). The concept was first articulated in Denis Diderot’s 1769 essay “Regrets on Parting with My Old Dressing Gown”. Diderot describes how, after purchasing a luxurious dressing gown, his other possessions appeared mismatched, leading him to replace them all, which ultimately caused regret. The purchase of a single product triggers the desire to acquire complementary items that align with it, giving rise to the Diderot effect. McCracken (1988) emphasizes that this effect is tied to aesthetic concerns. Identity alignment, status-seeking, and the need for belonging are psychological mechanisms that reinforce these concerns (McCracken, 1988).

In digital consumption, the Diderot effect becomes more pronounced (Song, 2023). This is because “behavioral architecture” (Thaler & Sunstein, 2008) makes complementary products more visible. Easy and fast payment options reduce the pain of spending (Prelec & Simester, 2001). One-click shopping, “Buy Now Pay Later” options, and cross-selling suggestions in the shopping cart accelerate decision-making (Prelec & Simester, 2001; Singh & Sahni, 2024; Verhoef et al., 2007). Digital platforms also normalize the “complete set” appearance, while social proof mechanisms such as “customers who bought this also bought” or “celebrity recommendations” stimulate complementary purchases. Algorithmic recommendation systems strengthen the perception of an incomplete set by offering personalized products (Gallin & Portes, 2024). All these factors reinforce impulsive buying tendencies. For instance, when purchasing a dress online, being immediately presented with matching bag or shoe options, or repeatedly seeing complementary product advertisements on social media based on past searches and browsing, exemplifies this mechanism.

The sense of “mismatch” that arises after acquiring a new product is often resolved through sudden and unplanned complementary purchases (Çakaröz et al., 2022; Rook & Fisher, 1995). Çakaröz et al. (2022) demonstrated the role of the Diderot effect in unplanned purchases. Ergenekon Arslan (2022) linked the same mechanism to compulsive buying. These findings indicate that the Diderot effect is not limited to unplanned or compulsive purchases but may also fuel impulsive buying.

### 2.3. Impulsive Buying

Impulsive buying emerges from a strong and sudden urge. The purchased product is often not on the individual’s initial shopping list, and at the moment of decision, sufficient attention may not be given to price or utility evaluation (Qureshi et al., 2025; Y. Shi & Joo, 2023). In online shopping environments, this behavior becomes more prominent. Advertisements, promotional campaigns, personalized recommendations, and normative influences on social media can increase impulsive buying (Zafar et al., 2021).

Classical studies emphasize the spontaneous and emotional aspects of impulsive buying (Hausman, 2000; Rook, 1987) and note that this behavior can also be shaped by normative influences (Kwak et al., 2006; Rook & Fisher, 1995). However, impulsive buying cannot be explained solely by these factors. Rapid emotion regulation, hedonic compensation, and the search for instant gratification are also among its fundamental drivers. This process often results in overspending and regret (Chan et al., 2017; R. Shi et al., 2024; Verplanken et al., 2005). Shopping after a stressful day may provide temporary relief, but the effect is short-lived. Similarly, positive moods can also trigger impulsive buying. Self-reward, celebration, novelty seeking, social conformity, belongingness, or the fear of missing out are among the motives that further increase this behavior (R. Shi et al., 2024; Verplanken & Herabadi, 2001). Thus, impulsive buying emerges as a multidimensional behavior fueled by both negative and positive emotional processes.

This diversity has led to the examination of impulsive buying across different dimensions in the literature. Studies have conceptualized impulsive buying in cognitive (lack of planning, insufficient deliberation), emotional (pleasure seeking, excitement), and behavioral (sudden and uncontrolled purchases) dimensions (Muruganantham et al., 2013; Youn & Faber, 2000). Nevertheless, recent research has shown that examining this construct as a unidimensional phenomenon yields simpler and more consistent results (Badgaiyan et al., 2016; Iyer et al., 2020). In this study as well, impulsive buying is treated as a unidimensional construct. However, cultural context may also play a role, as motivations are likely to differ across individualistic and collectivist societies (P. Sharma et al., 2010).

### 2.4. Research Model and Hypotheses

This study tests the relationships among materialism, the Diderot effect, and impulsive buying. The starting point is the assumption that impulsive buying among highly materialistic individuals may be triggered by the Diderot effect. The central question is as follows: how does the desire to purchase a single product evolve into a chain of acquisitions? By addressing this question, the study tackles a theoretical gap in the literature while also identifying critical points in digital marketing that may lead to overconsumption. The focus of the study is the mediating role of the Diderot effect in this relationship. In addition, the model examines the direct effects of materialism on the Diderot effect and impulsive buying, as well as the indirect effects that emerge through the Diderot effect.

In the literature, the direct effect of materialism on the Diderot effect has been tested only to a limited extent. However, the conceptualization of the Diderot effect in terms of status/fit dimensions (Bozyiğit, 2025) and the demonstrated positive relationship between prestige sensitivity and the Diderot effect (Çakır & Eser, 2023) indicate that this relationship rests on a theoretical foundation. Materialistic values lead individuals to perceive possessions not merely as functional tools but also as markers of identity and status (Belk, 1984; Richins & Dawson, 1992). Thus, when a new product is acquired, a sense of “mismatch” may arise with existing possessions, prompting individuals to seek complementary products to resolve this inconsistency. Indeed, conceptual discussions have noted that materialistic tendencies may reinforce this “completion” orientation (Bozyiğit, 2025; Ergenekon Arslan, 2022). Furthermore, Ergenekon Arslan (2022), who examined materialism as a multidimensional construct, found that the success dimension of materialism significantly increases the Diderot effect.

To explain this relationship, theories that address consumers’ search for coherence among products can be instructive. Self-congruity theory (Sirgy, 1982) highlights that consistency between self-image and product image influences purchase decisions. The brand–self connection approach (Escalas & Bettman, 2005) emphasizes that individuals form symbolic bonds with brands, which are strengthened when aligned with self-image. The consumption constellation perspective (Englis & Solomon, 1996) shows that products are often perceived as complementary clusters. Taken together, these three approaches suggest that materialism not only fosters the desire to possess but also fuels the search for coherence among possessions, thereby strengthening the Diderot effect.

**H1.**  *Materialism positively influences the Diderot effect.*

Findings that materialism amplifies the Diderot effect bring attention to how this effect shapes shopping behaviors. When a new product is acquired, existing possessions may appear mismatched; this sense of inconsistency triggers unplanned purchases through the “completion of the set” motive (Çakaröz et al., 2022). This mechanism is also consistent with theoretical approaches that highlight consumers’ search for coherence among their possessions (Englis & Solomon, 1996; Sirgy, 1982).

Studies directly examining the effect of the Diderot effect on impulsive buying are limited. However, Santos et al. (2025) empirically confirmed this relationship. Beyond that, findings that complementary products trigger unplanned shopping (Çakaröz et al., 2022) and that the mechanism is associated with compulsive buying (Arslan & Bakır, 2023) underscore its significance. It has also been noted that the effect is not observed uniformly across all contexts. For example, Gürdin (2020) reported that the Diderot effect did not have a significant impact on classical purchasing decisions. In digital environments, however, the conditions differ. Recommendation systems highlight complementary products (Gallin & Portes, 2024; Song, 2023), and interface designs subtly steer decisions (Thaler & Sunstein, 2008). Easy payment options reduce the “pain of paying” (Prelec & Simester, 2001), accelerating purchasing processes. Moreover, emotional arousal and social cues such as “customers who bought this also bought” further strengthen impulsive buying tendencies (Rook & Fisher, 1995). Altogether, these findings make the role of the Diderot effect in impulsive buying more apparent.

**H2.**  *The Diderot effect positively influences impulsive buying behavior.*

In the literature, the effect of materialism on consumer behavior has long been discussed, with particular emphasis on its relationship with impulsive and compulsive buying tendencies. Studies conducted in various cultural and consumption contexts have shown that materialism strengthens impulsive buying tendencies (Atulkar & Kesari, 2018; Ningtyas & Vania, 2022; Pupelis & Šeinauskienė, 2023; Raj et al., 2024). In the Turkish context, it has been reported that certain dimensions of materialism (success and centrality) increase impulsive buying, while the happiness dimension reduces it (Açan et al., 2021). On the other hand, some research has demonstrated that materialistic values also predict compulsive buying behavior (Barbieri et al., 2025; Dittmar, 2005; Ellyawati et al., 2024; Pellegrino et al., 2022). Taken together, these findings suggest that materialism reinforces consumer behavior through different mechanisms and may have a direct effect on impulsive tendencies.

**H3.**  *Materialism positively influences impulsive buying behavior.*

Materialism strengthens the desire to close the gap between the “ideal self” and the “actual self.” How this desire is met in consumption is explained through the frameworks of self-discrepancy and compensatory consumption (Higgins, 1987; Rucker & Galinsky, 2008). The self-discrepancy approach suggests that this gap will be symbolically filled with complementary items that provide a sense of alignment (Higgins, 1987; Mandel et al., 2017). Similarly, the compensatory consumption literature shows that identity and status deficiencies are compensated through complementary acquisitions (Rucker & Galinsky, 2008). Therefore, individuals are inclined to collect pieces they perceive as consistent with their identity and as parts of a whole. This orientation is reinforced by social and cognitive signals. Status signaling and symbolic consumption turn the “complete set” appearance into a strong indicator of identity in the external world (Spence, 1973; Veblen, 2017). Social proof and normative conformity transform this appearance into expected standards (Cialdini & Goldstein, 2004). At the cognitive level, the means–end chain and schema congruity reveal that consistent sets are processed more fluently in the mind, accelerating the choice of complementary items (Gutman, 1982). In line with this framework, studies show a link between Diderot-type set completion and unplanned/impulsive add-on purchases (Çakaröz et al., 2022). Taken together, materialism heightens the motivation to complete coherent sets; through the Diderot effect, this tendency shifts choices toward impulse buying.

**H4.**  *The Diderot effect mediates the relationship between materialism and impulsive buying behavior.*

**Figure 1:**
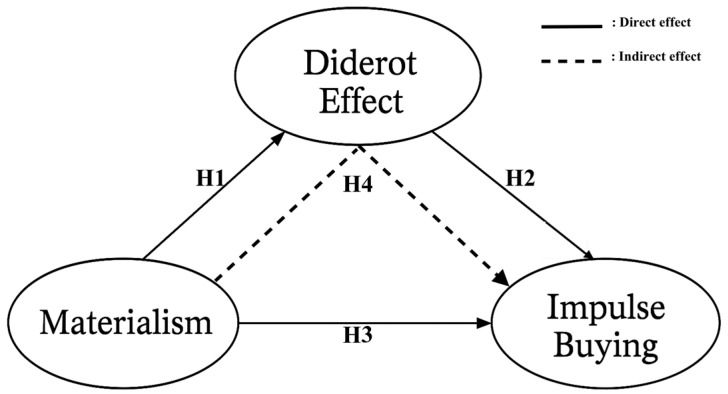
Research Model.

The theoretical model constructed based on the proposed relationships among the variables is presented in Figure 1. The model illustrates the effects of materialism on the Diderot effect and impulsive buying, as well as the mediating role of the Diderot effect.

Table 1, following the presented research model and hypotheses, enables quick comparison of the literature and compiles and summarizes the relevant studies. The table summarizes representative studies related to H1–H4, classifying evidence as Direct (empirical evidence directly testing the hypothesis), Partial/Analog (evidence closely related to the hypothesis, e.g., unplanned buying as a behavioral analog of impulsive buying), Theoretical (conceptual/theory-based support), Contextual (findings that strengthen or condition the effect under specific contexts), and Counterpoint (findings showing a null or weak effect).

## 3. Method

### 3.1. Research Design

This study is a cross-sectional and explanatory research conducted using a quantitative research method. The primary aim of the study is to empirically examine the effect of materialism on impulsive buying behavior in digital consumption environments and the mediating role of the Diderot effect in this relationship. The proposed research model was developed based on theoretical approaches within the consumer behavior literature, and the hypothesized relationships were empirically examined.

### 3.2. Pilot Study

In this study, a pilot test was carried out before the main data collection to see whether the model was feasible, to check the reliability and internal consistency of the measurement instruments, and to make sure the scales were understandable for participants and that the items worked consistently together.

At the beginning, the research model also included variables such as the Zeigarnik effect, social media usage, and unplanned buying, along with materialism, the Diderot effect, and impulsive buying. However, in the pilot study, some problems appeared in the subscales of these additional variables. In particular, the high correlation (r > 0.85) between unplanned buying and impulsive buying showed that there was no discriminant validity between the two. In addition, the reliability coefficients (e.g., Cronbach’s alpha < 0.70) of the Zeigarnik effect and social media usage subscales were found to be below the thresholds suggested in the literature. Therefore, the problematic variables were not included in the final model to keep it theoretically consistent and to ensure that the measurements were accurate and reliable.

The pilot study was carried out in January 2025 with 50 participants who were active social media users and familiar with the main concepts of the study. This number fits the range recommended in the literature for pilot studies, which is 30–50 participants (DeVellis, 2016; Johanson & Brooks, 2010). The results showed acceptable reliability for the three main constructs: Cronbach’s alpha was 0.721 for materialism, 0.751 for the Diderot effect, and 0.774 for impulsive buying, indicating that the measurement instruments were reliable.

In conclusion, the final model was revised and restructured around three core variables: materialism, the Diderot effect, and impulsive buying.

### 3.3. Sample and Data Collection

The research sample comprises adults aged 18 and above who are active users of social media platforms. Participants were selected through purposive sampling, taking into account their familiarity with key concepts related to consumer behavior. Since this study particularly aims to measure consumer behaviors emerging in digital environments, the data collection process was conducted online. This selection ensured the inclusion of individuals with behavioral characteristics and knowledge levels appropriate for the research objective (Darrat et al., 2016; Islam et al., 2022; Pradhan et al., 2018). The eligibility of participants was confirmed through additional screening questions that assessed their awareness of consumer behavior and their online/offline shopping experience. Participants were recruited through social media announcements, where the online survey link was shared. They were provided with detailed information about the study and asked to respond to the questions by considering their experiences in digital environments. It was emphasized that they could withdraw at any time, and participation was entirely voluntary. Data collection took place between January and March 2025, yielding a total of 428 responses.

An a priori power analysis grounded in model fit indices (χ^2^ and Root Mean Square Error of Approximation (RMSEA)) was employed to estimate the required sample size, utilizing the G*Power 3.1 software. Assuming close fit (RMSEA = 0.06) and poor fit (RMSEA = 0.10), with a significance level of 5% (α = 0.05) and statistical power of 90% (1–β = 0.90), it was calculated that a minimum of 307 participants would be sufficient for a chi-square test with 101 degrees of freedom (Faul et al., 2009). This ensured that the study was conducted with an adequate sample size to provide reliable and statistically valid results.

During the data cleaning process, outliers were identified using the z-score method based on the ±3 threshold, and respondents with zero-variance answers were excluded. The procedure for identifying outliers was based on the method proposed by Osborne and Overbay (2004). As a result of this analysis, data from 12 participants were excluded, and the final analyses were conducted with 416 participants.

In addition to demographic information, participants provided responses to scales measuring materialism, the Diderot effect, and impulsive buying behavior. Participation in the study was voluntary, and each participant provided informed consent by reading the information sheet presented at the beginning of the survey. The research process was conducted in accordance with ethical principles and received approval from the Scientific Research and Publication Ethics Committee.

### 3.4. Measures

All measures used in this study were adapted from empirically validated and widely used scales in the consumer behavior literature. The materialism scale was adapted from Pradhan et al. (2018), the Diderot effect scale was adapted from Gürdin (2020), and the impulsive buying measure was adapted from Darrat et al. (2016), Islam et al. (2022) and Sneath et al. (2009). The original versions of the scales consisted of 5 items for materialism, 8 items for the Diderot effect, and 5 items for impulsive buying.

In the process of adapting the scales into Turkish, the translation and back-translation method was used. The semantic consistency of the items was verified through back-translation conducted by two independent bilingual experts. Identified discrepancies were resolved based on the feedback of three subject-matter experts, ensuring conceptual equivalence of the scales through literature review and expert evaluation.

During the confirmatory factor analysis (CFA), two items from the materialism scale and two items from the Diderot effect scale were removed due to low factor loadings and model fit issues. In the final analysis, materialism was measured with 3 items, the Diderot effect with 6 items, and impulsive buying with 5 items. All items were rated on a 5-point Likert-type scale ranging from 1 (Strongly Disagree) to 5 (Strongly Agree).

### 3.5. Data Analysis

The data analysis process was conducted using Statistical Package for the Social Sciences (SPSS) 23, Linear Structural Relations (LISREL) 8.80, and Analysis of Moment Structures (AMOS) 23 software. In the initial stage, descriptive statistics, data cleaning procedures, and internal consistency analyses (Cronbach’s alpha) were performed using SPSS. Outlier analysis was conducted, extreme values were excluded, and only complete responses were included in the analyses.

Exploratory Factor Analysis (EFA). Prior to CFA, we conducted an EFA on the pilot sample, using principal axis factoring with Promax rotation. Sampling adequacy was evaluated with the Kaiser–Meyer–Olkin (KMO) index (values ≥ 0.50 acceptable), and factorability was examined via Bartlett’s test of sphericity, which is expected to be significant (*p* < 0.05). Factor retention followed the eigenvalue-greater-than-one rule together with inspection of the scree plot. Items were flagged for review if they showed cross-loadings > 0.50 (Hair et al., 2019a).

Common Method Bias (CMB). We assessed potential CMB by (i) Harman’s single-factor test, (ii) a common latent factor (CLF) check at the CFA stage (Polas, 2025).

Mardia’s coefficient was used to evaluate multivariate normality. In addition, key assumptions required for data analysis were examined through various diagnostic tests.

Following the assessment of normality, the measurement and structural models were examined sequentially. CFA was performed using LISREL to assess the measurement model. Within the scope of CFA, the extent to which each latent variable was represented by its corresponding observed variables was examined; standardized factor loadings, t-values, and overall fit indices were reported. Convergent validity was evaluated by analyzing the significance of factor loadings, ensuring that the average variance extracted (AVE) exceeded 0.50, and that composite reliability (CR) was above 0.70. Discriminant validity was tested by comparing how well each construct is represented by its own indicators (square root of AVE) relative to its correlations with other constructs.

Following the evaluation of the measurement model, the structural model was tested using Structural Equation Modeling (SEM) with LISREL. The structural model, which includes the hypothesized relationships, was evaluated by examining path coefficients (β), t-values, and the explained variance (R^2^). Moreover, model validity was assessed through overall model fit indices. Additionally, to verify the presence of a mediation effect, indirect relationships were tested in IBM SPSS AMOS 23 using the bias-corrected (BC) bootstrap procedure.

This multi-stage analysis strategy empirically confirmed the measurement validity, structural consistency of the theoretical model, and the presence of the proposed mediation mechanism.

## 4. Results

### 4.1. Descriptive Statistics

A total of 416 participants were included in the study. Of these, 67% were female and 33% were male. The literature indicates that online survey studies often show a higher proportion of female participants compared to male participants (Becker, 2022). Therefore, the predominance of women in the sample may be considered a natural outcome of the online data collection method. Moreover, previous studies have found that impulsive buying behavior tends to be more prevalent among female consumers (Puspitayanti et al., 2022; M. Sharma et al., 2020; P. Sharma et al., 2010; Tifferet & Herstein, 2012). Accordingly, the high proportion of female participants in this study aligns with the research topic and provides a meaningful sample composition in terms of content relevance.

The age range of the participants was between 18 and 65 years. The absence of individuals over 65 years of age was not due to an exclusion criterion but rather the nature of online data collection. The literature suggests that older adults have relatively lower levels of access to digital platforms and lower participation rates in online surveys (Becker, 2022). This may explain why the sample was largely composed of individuals from younger and middle-aged groups.

In terms of occupational distribution, the largest group consisted of students (39.2%), followed by public sector employees (30.3%), private sector employees (17.3%), homemakers (4.6%), retirees (1.9%), and participants from other occupational groups (6.7%). Regarding marital status, 60% of the participants were single and 40% were married. The literature suggests that young individuals—particularly students and single participants—are more prone to impulsive buying behavior (M. Sharma et al., 2020). Therefore, the overrepresentation of students and single individuals in the sample aligns with the purpose of the study and provides meaningful representation for analyzing the consumption behaviors of the target group.

As for educational background, 44% of the participants held a bachelor’s degree, 18.5% a doctoral degree, 16.6% an associate degree, 14.4% a master’s degree, 5.5% a high school diploma, and 1% had completed primary education. Some participants appeared to indicate their current level of education rather than their completed degrees. Therefore, the high proportion of those reporting undergraduate education may reflect both current enrollment and completed qualifications. However, this information is presented solely to describe the sample profile, and no comparative analyses based on demographic variables were conducted in this study.

The participants’ levels of materialism ($\bar{X}$ = 2.62, SD = 0.78) and perception of the Diderot effect ($\bar{X}$ = 2.60, SD = 0.84) were below moderate levels. The tendency toward impulsive buying was even lower ($\bar{X}$ = 2.05, SD = 0.86). These findings indicate that participants generally exhibited low levels of materialism, low perception of the Diderot effect, and particularly low levels of impulsive buying tendencies. The standard deviations suggest a moderate degree of variability among participants.

### 4.2. Exploratory Factor Analysis and Common Method Bias

An initial EFA supported a three-factor solution consistent with the theorized constructs (Materialism, Diderot Effect, Impulsive Buying). Sampling adequacy was good (KMO = 0.635; Bartlett χ^2^(df = 91) = 388, *p* < 0.001). The three factors explained 62% of the total variance. All retained items loaded on their intended factors (Materialism: 0.61–0.78; Diderot Effect: 0.59–0.80; Impulsive Buying: 0.59–0.63). These results justified proceeding to CFA with the final 3-construct.

Harman’s single-factor test indicated that the first unrotated factor accounted for 39% of the variance, below the conventional benchmark for a dominant method factor. Adding a common latent factor (CLF) at the CFA stage did not materially improve fit; the CLF model showed poor fit (χ^2^/df = 11.46; RMSEA = 0.153). Taken together, these diagnostics suggest that common method bias is unlikely to threaten the validity of the findings.

### 4.3. Normality Test

Before proceeding with the SEM analysis, the multivariate normality of the dataset was examined. The results of the multivariate normality test indicated statistically significant values for both skewness (Z = 21.238, *p* < 0.001) and kurtosis (Z = 12.914, *p* < 0.001). Additionally, the total Mardia’s coefficient (617.845) confirmed that the dataset did not conform to a multivariate normal distribution.

Due to the violation of the normality assumption, the classical Maximum Likelihood (ML) estimation method was not deemed appropriate. Instead, the Robust Maximum Likelihood (RML) method, which is more resistant to deviations from normality, was employed. This approach enhances the validity of the results and provides more reliable estimates under non-normal data conditions (Kline, 2023).

### 4.4. Measurement Model

To assess the validity and reliability of the latent constructs used in the study, CFA was conducted. The measurement model included three latent variables—materialism, the Diderot effect, and impulsive buying—each represented by multiple observed items. Materialism was modeled as the independent variable, the Diderot effect as the mediating variable, and impulsive buying as the dependent variable. During the analysis, items with factor loadings below 0.50 (as predetermined for the Diderot effect construct) were removed from the model. This refinement improved the overall factor structure. The final CFA was conducted on a total of 14 items.

The fit of the measurement model was evaluated using LISREL 8.80 with the RML estimation method. The chi-square/degrees of freedom ratio was 2.87, indicating an acceptable model fit. Given the sensitivity of the chi-square test to sample size, additional fit indices were also examined. The Comparative Fit Index (CFI = 0.97), Non-Normed Fit Index (NNFI = 0.97), Normed Fit Index (NFI = 0.96), Incremental Fit Index (IFI = 0.97), and Goodness-of-Fit Index (GFI = 0.92) values all exceeded the recommended threshold of 0.90, indicating good model fit (Bagozzi & Yi, 1988; Bentler & Bonett, 1980; Bollen, 1989; Jöreskog & Sörbom, 1996). The Standardized Root Mean Square Residual (SRMR = 0.047) reflected low residual error and strong alignment with the observed data (Hu & Bentler, 1999). The RMSEA value of 0.067 was below the acceptable threshold of 0.08, indicating an overall acceptable model fit (Schermelleh-Engel et al., 2003). These findings support the adequacy of the model’s overall validity.

Internal consistency of the scales was assessed using Cronbach’s alpha. The reliability coefficients were 0.713 for materialism, 0.874 for the Diderot effect, and 0.831 for impulsive buying—all above the commonly accepted threshold of 0.70 (George & Mallery, 2003). The overall Cronbach’s alpha for the entire measurement instrument was calculated as 0.878, indicating strong internal consistency.

The evaluation of validity was conducted based on convergent and discriminant validity criteria. For convergent validity to be established, factor loadings must exceed 0.50 and be statistically significant; the AVE should be greater than 0.50; and CR should exceed 0.70 (Hair et al., 2019b). According to CFA results, all factor loadings ranged from 0.59 to 0.83 and were all statistically significant.

CR values were calculated as 0.72 for materialism, 0.88 for the Diderot effect, and 0.83 for impulsive buying. The AVE values were 0.46 for materialism, 0.59 for the Diderot effect, and 0.50 for impulsive buying. Although the AVE value for materialism fell slightly below the 0.50 threshold, the construct was deemed acceptable due to its adequate CR value and significant factor loadings (Fornell & Larcker, 1981).

Examining Table 2 indicates that discriminant validity is achieved under the Fornell–Larcker criterion. The AVEs exceed the corresponding shared variances (off-diagonal R^2^). Furthermore, none of the correlation values exceeded 0.85, suggesting an absence of multicollinearity risk. These findings reinforce the distinctiveness of the constructs and validate the structural integrity of the measurement model. The factor loadings and CFA results are presented in Table 3.

### 4.5. Structural Model

The structural model was tested using LISREL 8.80 with the RML estimation method. The chi-square to degrees of freedom ratio (χ^2^/df) was 2.87, indicating an acceptable level of model fit. Given the sensitivity of the chi-square statistic to sample size, alternative fit indices were also considered. The CFI (0.97), NNFI (0.97), NFI (0.96), IFI (0.97), and GFI (0.92) values all exceeded the accepted benchmark of 0.90, confirming an adequate fit of the model to the data (Bagozzi & Yi, 1988; Bentler & Bonett, 1980; Bollen, 1989; Jöreskog & Sörbom, 1996). Additionally, the SRMR value (0.047) and the RMSEA value (0.067) were both within acceptable limits, further confirming the adequacy of the model fit (Hu & Bentler, 1999; Schermelleh-Engel et al., 2003). These findings demonstrate that the structural model exhibits satisfactory overall fit.

Hypothesis testing results revealed that materialism had a significant and positive effect on both the Diderot effect (β = 0.50, t = 6.88) and impulsive buying behavior (β = 0.26, t = 2.94). Additionally, the Diderot effect had a significant positive effect on impulsive buying behavior (β = 0.47, t = 5.65). The model explained 25% of the variance in the Diderot effect (R^2^ = 0.25) and 39% of the variance in impulsive buying behavior (R^2^ = 0.39), indicating a moderate explanatory power.

Mediation analysis was conducted using the BC bootstrap method in IBM SPSS AMOS 23, based on 5000 resamples and 95% confidence intervals. According to the results, the direct effect of materialism on the Diderot effect was statistically significant (β = 0.31, CI [0.17, 0.45], *p* < 0.001). The Diderot effect also had a strong and significant effect on impulsive buying (β = 0.60, CI [0.51, 0.69], *p* < 0.001). The direct effect of materialism on impulsive buying (c′) remained significant (β = 0.44, CI [0.28, 0.61], *p* < 0.001). Furthermore, the indirect path from materialism to impulsive buying through the Diderot effect was also found to be significant (β = 0.20, CI [0.11, 0.28], *p* < 0.001). According to the framework proposed by Zhao et al. (2010) these findings indicate the presence of partial mediation.

**Figure 2:**
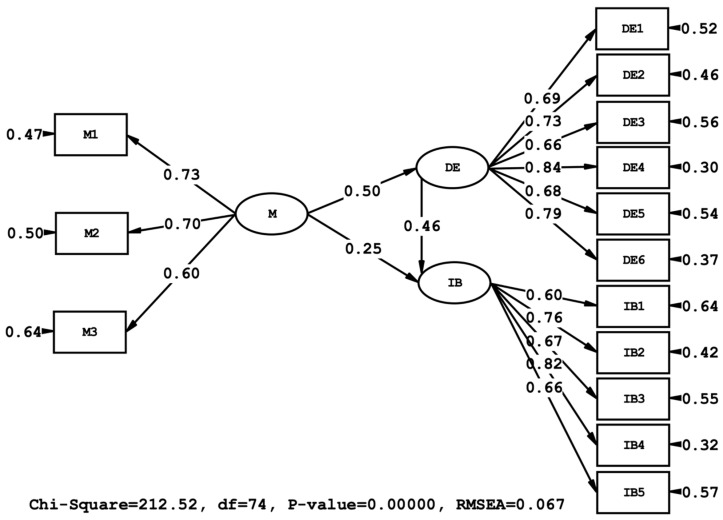
Path Diagram of Model.

The results of the hypothesis tests are presented in Table 4, and the path diagram of the structural model is shown in Figure 2.

## 5. Conclusions

This study examined the influence of materialism on impulsive buying and the mediating role of the Diderot effect in this relationship. The analysis revealed that materialism significantly and positively affects both the Diderot effect and impulsive buying. This shows its partial mediating role and highlights the importance of psychological mechanisms in shaping consumer decisions.

### 5.1. Discussion

In this study, materialism was examined as a unidimensional construct, and it was found to significantly increase the Diderot effect. Ergenekon Arslan (2022) approached materialism as a multidimensional construct and reported that the success dimension of materialism enhances the Diderot effect. Similarly, Çakır and Eser (2023) showed that prestige sensitivity, which is associated with materialistic values, further strengthens this effect. Therefore, the findings can be considered partially consistent with previous studies.

This study also demonstrates that the Diderot effect strongly triggers impulsive buying behavior. Although studies directly examining the effect of the Diderot effect on impulsive buying are limited, the results are consistent (Santos et al., 2025). Moreover, findings that complementary products stimulate unplanned shopping (Çakaröz et al., 2022) and are linked to compulsive shopping (Arslan & Bakır, 2023) also align with the results of this study. By contrast, Gürdin (2020) concluded that the Diderot effect does not have a significant impact on classical purchase decisions. In digital environments, however, conditions differ: faster purchases (Santos et al., 2025) and recommendation systems highlighting complementary products (Song, 2023) amplify the Diderot effect. Thus, the emergence of the Diderot effect as a unique psychological mechanism that triggers impulsive buying, when considered alongside studies showing that other psychological processes also influence impulsive shopping behavior (Gallin & Portes, 2024; Li et al., 2025; Y. X. Xia et al., 2024), contributes to a broader understanding of the phenomenon.

This study further finds that materialistic tendencies moderately increase the desire for impulsive shopping. This result is consistent with findings from different cultural contexts (Atulkar & Kesari, 2018; Ningtyas & Vania, 2022; Pupelis & Šeinauskienė, 2023; Raj et al., 2024). Examining this relationship in the Turkish context is particularly valuable, as the rapid increase in internet usage (TÜİK, 2024) suggests that impulsive shopping may eventually lead to uncontrollable consumption cycles (compulsive buying) (Barbieri et al., 2025; Faraz & Anjum, 2025). Findings from the Turkish sample contribute to understanding the transformation of consumer culture in developing societies. Studies investigating the relationship between materialism and consumer culture in Turkey (e.g., Avcı, 2023; Yılmaz et al., 2025) appear limited in scope. Açan et al. (2021) reported that certain dimensions of materialism (success and centrality) increase impulsive buying, while the happiness dimension decreases it. It has also been shown that materialistic values influence compulsive buying behavior (Barbieri et al., 2025; Dittmar, 2005; Ellyawati et al., 2024; Ergenekon Arslan, 2022; Pellegrino et al., 2022). Although the focus of this study is impulsive buying, the findings show partial similarities.

The main contribution of this study is to reveal the Diderot effect as a mediating mechanism in the relationship between materialism and impulsive buying. While the Diderot effect has been conceptually discussed (McCracken, 1988; Tokmak, 2019) and examined in the context of digital consumption (Çakaröz et al., 2022; Santos et al., 2025), its mediating role has not been explicitly tested; this study fills that gap. When materialistic desires combine with the search for coherence triggered by the Diderot effect, consumers’ impulsive tendencies emerge much more strongly.

Empirical findings quantitatively corroborate this framework. The mediation estimates indicate that a substantial share of the effect operates through the Diderot effect: the indirect effect is β = 0.20 (95% CI [0.11, 0.28]), while the direct path remains significant (c′ = 0.44); this implies that approximately 31% of the total effect (0.20/0.64) is transmitted via the mediator. These figures quantify the mechanism’s contribution within the overall pathway.

### 5.2. Managerial Implications

Empirical findings indicate that a substantial share of the total effect (31%) is transmitted through the mediating mechanism. As materialistic orientation increases, the Diderot effect becomes more salient and consumers gravitate toward complementary items, which in turn triggers impulsive buying. Online recommendation systems further amplify this tendency (Fadilah et al., 2025; Song, 2023). However, strategies that merely push consumers to buy more are not sustainable (Adkisson, 2008; Mathur et al., 2019). Accordingly, a framework is needed that balances tactics promoting additional purchases with user welfare and trust. The aim is to manage the Diderot effect and reduce impulsive buying. Within this scope, the following recommendations are presented.

Recommendation intensity should be set with clear thresholds (e.g., per-session rate limits), and exposure to recommendation systems should be kept below the saturation point. Alongside options like “one-click” payment, a visible standard payment path should also be available. A brief cooling-off period should be defined in the cart; for higher totals, there should be budget alerts and a “save for later” option. Before payment, the total cost (including taxes/shipping/fees) should be shown transparently. Opt-out (e.g., “turn off recommendations,” unsubscribe from emails, disable personalization) and preference-persistence settings should be easy to access.

Basing marketing and recommendation strategy solely on Average Order Value is insufficient. In addition, return/complaint rates, repeat purchase (retention) rates, opt-out signals, and Net Promoter Score should be tracked (Farris et al., 2010; Reichheld, 2003). In practice, attach rate (the share of orders that add complementary products) and planned A/B tests (A = current, B = new design) are also good indicators (Kohavi et al., 2009). If attach rate rises while NPS falls or returns/complaints increase, the recommendation strategy should be softened (Kohavi et al., 2009).

A short roadmap should be created with the relevant teams. In this way, recommendation intensity, transparency, the opt-out process, budget alert thresholds, and boundaries for vulnerable groups are clear from the outset. Responsible-recommendation training should be provided to the design teams. Cancel/undo flows should be easy to execute. In practice, in identity-visible categories (e.g., apparel, phone cases), local/recycled complements can be highlighted; for price-sensitive users, budget reminders can be set as the default. In this way, impulsive add-on purchases decrease, decision quality improves, and perceptions of autonomy, fairness, and trust are preserved (Johnson et al., 2012; Mathur et al., 2019; Thaler & Sunstein, 2008; L. Xia et al., 2004).

In sum, when the integrated model with the mediating mechanism is put into practice under these recommendations, impulsive buying can be contained—balancing short-term revenue with long-term trust and sustainability.

### 5.3. Societal and Individual Implications

Consumption is not merely the outcome of individual choices; it takes shape within the habits, shared meanings, and everyday interactions of social life, gradually becoming a habit (Kennedy et al., 2015; Warde, 2005; Weder et al., 2025). While it is important for people to build their own awareness, this cannot be left only to individuals themselves. Consumer associations, non-governmental organizations, and public institutions can guide this process. Awareness projects, educational activities, or social media campaigns could help people see more clearly that their purchasing behavior is driven by psychological mechanisms such as materialism and the Diderot effect. For instance, showing with simple examples how the urge to buy complementary products arises after purchasing a single item could make these mechanisms more tangible. With this kind of effort, individuals could both protect their budgets and personal well-being, and at the same time contribute to the development of a more sustainable culture of consumption.

### 5.4. Limitations and Suggestions for Future Research

This study has certain limitations. First, although the proposed model revealed meaningful and explanatory relationships, the proportion of explained variance remained at a moderate level (39%). This indicates that other psychological mechanisms or social and environmental factors may also influence impulsive buying. Therefore, future studies could examine the impact of variables such as social media use, social norms, algorithmic recommendation systems, financial awareness, sustainable consumption, and ethical consumption in a multidimensional framework.

Second, the cultural and socioeconomic characteristics of the sample may limit the generalizability of the findings. Thus, testing the model in different cultural settings and conducting comparisons between countries at varying levels of development could provide a deeper understanding of cultural influences.

Finally, this study is cross-sectional. Longitudinal or experimental research designs could help assess the temporal dynamics of the relationships identified in this study. Future studies could experimentally investigate how the Diderot effect is triggered in digital marketing environments, providing both theoretical and practical contributions.

## Figures and Tables

**Table 1:** Representative studies and their linkage to H1–H4.

Theory/Model	Study (Year)	Focus	Key Finding	Hypothesis
Materialism → Diderot effect	Ergenekon Arslan (2022)	Materialism (dimensions), Diderot	The ‘success’ facet of materialism significantly increases the Diderot effect.	H1 (direct)
Prestige sensitivity → Diderot effect	Çakır and Eser (2023)	Prestige sensitivity, Diderot	Prestige sensitivity strengthens the Diderot effect.	H1 (proximal)
Conceptual framing of Diderot (status/fit)	Bozyiğit (2025)	Status and fit dimensions of Diderot	Conceptually positions Diderot through status/fit alignment.	H1 (theoretical)
Diderot effect → Impulsive buying	Santos et al. (2025)	Diderot effect, impulsive buying	Reports a positive effect of the Diderot effect on impulsive buying (early evidence).	H2 (direct)
Diderot effect → Unplanned purchase	Çakaröz et al. (2022)	Diderot effect, unplanned buying	Diderot effect is positively associated with unplanned purchases (analog to IB).	H2 (partial/analog)
Diderot-like mechanism ↔ Compulsive buying	Arslan and Bakır (2023)	Diderot mechanism, compulsive buying	Completion-like mechanism relates to compulsive tendencies (heavier form of IB).	H2 (partial/analog)
Consumption constellations (sets)	Englis and Solomon (1996)	Complementary sets	Products are perceived/consumed as complementary sets; facilitates completion.	H2 (theoretical)
Normative/social triggers of impulse	Rook and Fisher (1995)	Norms and impulses	Normative influences trigger spur-of-the-moment buying; reinforces completion-driven choices.	H2 (supportive)
Context variability/null finding	Gürdin (2020)	Diderot, classical purchase	No significant effect in classical contexts; highlights contextual dependence.	H2 (counterpoint)
Materialism → Impulsive buying	Atulkar and Kesari (2018)	Materialism, IB	Materialism positively predicts impulse buying.	H3 (direct)
Materialism → Impulsive buying	Pupelis and Šeinauskienė (2023)	Materialism, IB	Positive and significant link across a European sample.	H3 (direct)
Materialism → Impulsive buying	Raj et al. (2024)	Materialism, IB	Materialism has a positive effect on impulse buying in recent data.	H3 (direct)
Materialism ↔ Impulsive/compulsive buying	Dittmar (2005); Dittmar and Bond (2010)	Materialism, IB/CB	Materialism associates with impulsive/compulsive tendencies.	H3 (broad support)
Mediation path: M → D → IB (conceptual)	Belk (1984); Richins and Dawson (1992)	Identity/status orientation	Materialism heightens identity/status motives that can foster set-coherence seeking.	H4 (theoretical)
Coherent sets and self-congruity	Englis and Solomon (1996); Sirgy (1982); Escalas and Bettman (2005)	Sets; self–brand links	Coherent sets and self-congruity guide complementary choices; aligns with mediation logic.	H4 (theoretical)

Notes: M = Materialism; D = Diderot effect; IB = Impulsive Buying; CB = Compulsive Buying.

**Table 2:** Discriminant validity analysis (Fornell–Larcker Criterion).

Construct	Materialism	Diderot Effect	Impulse Buying
Materialism	(0.46)		
Diderot Effect	0.25	(0.59)	
Impulse Buying	0.23	0.35	(0.50)

Diagonal entries (in parentheses) report AVEs; off-diagonal cells report squared inter-construct correlations (R^2^).

**Table 3:** Measurement Items and CFA Results.

Construct	Item	Standardized Loading	t-Value	CR	AVE
Materialism	I prefer to make a good impression by purchasing expensive products.	0.73	11.25	0.72	0.46
	I believe that people who own expensive items are admired in society.	0.70	15.35		
	I think material possessions are important indicators of success in life.	0.60	11.85		
Diderot Effect	If one of my items does not match the others, I replace it with a new one.	0.70	11.89	0.88	0.59
	I keep replacing my belongings until they all match harmoniously.	0.73	13.90		
	Incomplete or unfinished things lead me to buy more.	0.67	12.18		
	Mismatched items among my belongings lead me to shop.	0.83	14.76		
	Missing complementary parts of any item encourage me to purchase.	0.68	11.89		
	When I buy something new, if it doesn’t fit with the old ones, it motivates me to buy.	0.79	13.90		
Impulse Buying	I want to buy something regardless of what it is.	0.59	8.34	0.83	0.50
	I purchase items to feel better emotionally.	0.76	9.34		
	I notice that I buy items beyond my financial means.	0.68	9.16		
	I shop to distract myself from mental preoccupations.	0.82	9.60		
	I buy things that I don’t use after purchasing.	0.65	10.64		

**Table 4:** Results of Hypothesis Testing.

Hypothesis	Path	β	t/CI	Result
H1	Materialism → Diderot Effect (Direct Effect)	0.50	6.88	Supported
H2	Materialism → Impulse Buying (Direct Effect)	0.25	2.94	Supported
H3	Diderot Effect → Impulse Buying (Direct Effect)	0.46	5.65	Supported
H4	Materialism → Diderot Effect → Impulse Buying (Indirect Effect)	0.20	CI [0.11, 0.28]	Supported

## Data Availability

The dataset generated and analyzed during this study is available from the author upon reasonable request due to ethical restrictions.

## Abbreviations

The following abbreviations are used in this manuscript:

SEM	Structural Equation Modeling
CFA	Confirmatory Factor Analysis
AVE	Average Variance Extracted
CR	Composite Reliability
RMSEA	Root Mean Square Error of Approximation
CFI	Comparative Fit Index
NNFI	Non-Normed Fit Index
NFI	Normed Fit Index
IFI	Incremental Fit Index
GFI	Goodness-of-Fit Index
SRMR	Standardized Root Mean Square Residual
ML	Maximum Likelihood
RML	Robust Maximum Likelihood
BC	Bias-Corrected (bootstrap)
SPSS	Statistical Package for the Social Sciences
LISREL	Linear Structural Relations
AMOS	Analysis of Moment Structures
df	Degrees of Freedom
EFA	Exploratory Factor Analysis
KMO	Kaiser–Meyer–Olkin
CMB	Common Method Bias
CLF	Common Latent Factor
